# Wilhelm His Senior und die Entwicklung der Paraffineinbettung

**DOI:** 10.1007/s00292-021-00943-8

**Published:** 2021-05-13

**Authors:** Tim van der Lem, Merijn de Bakker, Gerhard Keuck, Michael K. Richardson

**Affiliations:** 1 Institute of Biology, Sylvius Laboratory, Leiden University (IBL), Sylviusweg 72, 2333 BE Leiden, Niederlande; 2 Geißspitzweg 8, 65929 Frankfurt, Deutschland

**Keywords:** Hühnerembryo, Histologie, Mikrotom, Histopathologie, Gewebeeinbettung, Chick embryo, Histology, Microtome, Histopathology, Tissue embedding

## Abstract

Die paraffinbasierte histologische Untersuchung ist eines der wichtigsten angewendeten Laborverfahren in der diagnostischen Histopathologie. Die Entwicklung der Paraffineinbettung wird oft dem Pathologen Edwin Klebs zugeschrieben. Klebs orientierte sich an den Arbeiten von Stricker, der Embryonen in eine Mischung aus heißem Tristearin und weißem Bienenwachs einbettete, und experimentierte mit Paraffin, um Tumorgewebe einzubetten. Er verwarf den Ansatz aber rasch, da Paraffin das Gewebe nicht infiltrierte. Klebs stand im Briefwechsel mit dem Embryologen Wilhelm His Senior, der von dessen Experimenten erfuhr und beschloss, sich an der Paraffineinbettung zu versuchen. His entwässerte Hühnerembryonen in Alkohol, klärte sie mit Lavendelöl und träufelte heißes Paraffin darüber. Dieses Verfahren ermöglichte ihm die Anfertigung guter Schnitte. In der vorliegenden Arbeit haben wir His’ Protokoll der Paraffineinbettung nachvollzogen, um zu ermitteln, ob His tatsächlich die Infiltrationseinbettung mit Paraffin gelungen ist. Wir befolgten das Protokoll aus seiner 1868 erschienenen Monografie. Anhand der Anweisungen von His gelang es uns nicht, Schnitte mit der von ihm illustrierten Qualität herzustellen. In der Regel zerfiel das Gewebe beim Schneiden wegen schlechter Wachsinfiltration. Brauchbare Schnitte wurden nur bei Abwandlung von His’ Protokoll durch das Wiedereinschmelzen der eingebetteten Embryos in frischem Paraffin erreicht. Möglicherweise ist es uns nicht gelungen ist, das Protokoll von His detailgetreu zu replizieren, oder das Protokoll war unvollständig. Nach unserer Einschätzung hat His wahrscheinlich die Infiltrationseinbettung mit Paraffin erfunden und perfektioniert, aber kein vollständiges Protokoll veröffentlicht.

**Zusatzmaterial online:**

Zusätzliche Informationen „Das histologische Verfahren von His“ Originalfassung aus [15] sind in der Online-Version dieses Artikels (10.1007/s00292-021-00943-8) enthalten.

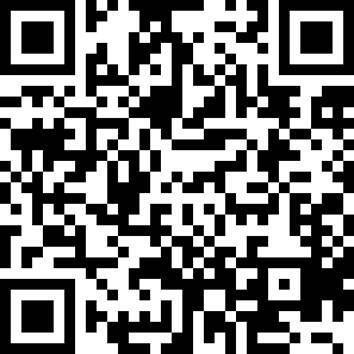

## Abstract (en)

Paraffin histology is one of the most important and commonly used laboratory techniques in diagnostic histopathology. The discovery of paraffin embedding is often attributed to the pathologist Edwin Klebs. Klebs was following the lead of Stricker, who embedded embryos in a mixture of hot stearin and white beeswax. We show that Klebs experimented with paraffin wax for embedding tumour tissue. But he quickly rejected it as unsuitable because paraffin wax did not infiltrate the tissue. One of Klebs’ correspondents, embryologist Wilhelm His, Sr., learned of Klebs’ experiments and decided to try paraffin embedding. His dehydrated chicken embryos in alcohol, cleared them in lavender oil, and dripped hot paraffin wax onto them. This process allowed His to cut good sections. Here, we have replicated His’s paraffin embedding protocol in order to determine whether His had indeed made the landmark discovery of infiltration embedding with paraffin wax. We followed the protocol that he gives in his 1868 monograph on the early development of the chicken. The protocol described by His failed, in our hands, to yield sections of the quality that he illustrates in his monograph. Typically, the tissue disintegrated when sectioned due to poor infiltration of the wax. Usable sections could only be obtained if His’s protocol was modified by melting the embedded embryos in fresh paraffin wax. One explanation for our findings is that we failed to faithfully replicate His’s protocol. Another is that his protocol was incomplete. We suggest that His is likely to have discovered and perfected infiltration embedding with paraffin wax but did not publish a complete protocol.

## Hintergrund

Das Verfahren der Einbettung dient der Vorbereitung von Gewebeproben für die mikroskopische Untersuchung. Dabei wird die Probe in eine feste Substanz eingebracht, um sie mit einem Mikrotom schneiden zu können [22]. Die Substanz sollte hart genug sein, um das Gewebe zu stützen, aber auch weich genug, um eine einfache Schnittführung zu erlauben. Es gibt 2 Arten der Einbettung [7]. Bei der peripheren Einbettung wird das Gewebe lediglich umschlossen und nur von außen gestützt. Die Infiltrationseinbettung oder interstitielle Einbettung dagegen stützt das Gewebe von außen und innen, da das Einbettungsmedium es vollständig durchdringt. Die Infiltration kann ein Intermedium erfordern, das heißt ein Lösungsmittel, das sowohl mit dem Alkohol für die Gewebeentwässerung als auch mit dem Einbettungsmedium mischbar ist [7, 21, 22]. Viele Intermedien wirken auch als Klärmittel, die das Gewebe optisch transparent machen [22, S. 68].

Ein weit verbreitetes Einbettungsmedium ist Paraffin. Paraffin wird aus Erdöl gewonnen und besteht aus einer Mischung von gerad- und verzweigtkettigen Kohlenwasserstoffen [37]. In Alkohol ist es schlecht löslich [30, S. 356], weshalb es für die Infiltrationseinbettung nur in Kombination mit einem Intermedium verwendbar ist. Eine der vielen nützlichen Eigenschaften von Paraffin besteht darin, dass die erhaltenen Dünnschnitte (5–7 µm) bei ihrer Anfertigung dazu neigen, in einem Band aneinander zu haften, sodass in einem Zug mehrere Schnitte auf den Objektträger verbracht werden können.

Die paraffinbasierte histologische Untersuchung findet heute so breiten Einsatz, dass sie nicht selten als histologischer „Standard“ bezeichnet wird [3, 26, 27]. Sie wird in der diagnostischen Histopathologie zur Untersuchung abnormer Zellen und Gewebestrukturen eingesetzt [32]. Zudem findet sie in vielen Bereichen der biomedizinischen Forschung Anwendung, wo Gewebestrukturen [26] und Genexpressionsmuster untersucht werden [24].

### Histologische Technik bis in die 1860er-Jahre

In den frühen 1860er-Jahren waren Botaniker schon lange in der Lage, histologische Schnitte anzufertigen. Frische Pflanzengewebe sind häufig ausreichend stabil für eine manuelle Schnittführung mit einer Rasierklinge [36]. Auch Mikrotome gab es bereits. Im Jahr 1770 beschrieb Hill ein von Cummings für das Schneiden von Holzgewebe entworfenes Mikrotom („cutting engine“, Schneidmaschine) [12]. Zur Befestigung der Probe während der Schnittführung klemmten Botaniker sie häufig zwischen Streifen eines weichen Stützmaterials, etwa aus dem Mark junger Holunderzweige (*Sambucus nigra*) [5].

Anders als pflanzliche Gewebe sind frische tierische und menschliche Gewebe in der Regel zu weich für die Anfertigung dünner Schnitte, weshalb sie gehärtet oder eingebettet werden müssen. Gewöhnlich wurden tierische Weichgewebe mit Alkohol oder einem Fixiermittel gehärtet [29, S. 460–473] oder man ließ sie zur Winterzeit im Freien gefrieren [38]. Geeignete Einbettungsverfahren gab es für tierische Gewebe noch nicht. Botaniker begannen aber, mit Einbettungsmedien zu experimentieren.

Laut einem anekdotischen Bericht hatte Eduard Fenzl „vor Jahren“ kleine Pflanzengewebestücke in Tristearin eingebettet, um sie für das Schneiden vorzubereiten [17, S. 11]. Laut Apáthy war unter Botanikern die Ansicht verbreitet, dass Fenzl auch Paraffin als Einbettungsmedium einführte [2, S. 80–81 Fußnote 3], aber auch dieser Bericht ist anekdotisch. Schatz empfahl, geschmolzenes Tristearin in trockene, mürbe Holzproben zu spritzen, um sie schneidbar zu machen [35, S. 66]. Tristearin ist ein Triglyzerid der Stearinsäure [41] und wurde zur damaligen Zeit in unreiner Form aus tierischen Fetten gewonnen [33, S. 52].

### 1864: Infiltrationseinbettung mit Bienenwachs und Tristearin

Salomon Stricker erkannte in seinen Studien zur Entwicklung von Fröschen (*Bufo* spp.), dass das Gewebe für eine hinreichende mikroskopische Untersuchung zu undurchsichtig war [39]. Daher entschloss er sich, mikroskopische Schnitte anzufertigen. Zuerst fixierte er die Embryonen und Larven mit Chromsäure, dann entwässerte und klärte er sie in reinem Alkohol und Terpentinöl. Durch dieses Verfahren wurde das Gewebe transparent [39, S. 53] – und das Terpentinöl wirkte vermutlich als Intermedium. Dann träufelte Stricker eine geschmolzene Mischung von weißem Wachs und Tristearin auf die geklärten Embryonen. Das „weiße Wachs“, wie er es nannte, war wahrscheinlich an der Sonne gebleichtes Bienenwachs [30, S. 603]. Schließlich fertigte Stricker Schnitte der Embryonen an (siehe seine Tafel I, [39]).

### 1867: Edwin Klebs und die periphere Einbettung mit Paraffin

Paraffin wurde von Edwin Klebs als Einbettungsmedium eingeführt [9, 30, 40]. Klebs, Professor für Pathologie an der Universität Bern, forschte an Larynxtumoren [18]. Er erkannte, dass die Histopathologie und deren Anwendung zur Untersuchung und Diagnostik von Tumorerkrankungen damals noch immer ein schwach entwickelter Wissenschaftszweig war. Mit der von ihm sog. Einschmelzungsmethode stellte er mikroskopische Schnitte der Tumoren her. Nach seiner Erinnerung wurde dieses Verfahren wahrscheinlich erstmals von Stricker in embryologischen Studien eingesetzt [18, S. 207–208 Fußnote]. Später korrigierte er sich jedoch [19, S. 164] und schrieb die Erfindung der „Einschmelzung“ Rudolf Heidenhain zu, der eine konzentrierte Gummi-arabicum-Lösung als Medium verwendete. Noch später erklärte Klebs, dass Heidenhain ihm gegenüber schriftlich verneint hätte, der Erfinder des Verfahrens zu sein [20, S. 206 Fußnote].

Klebs ersetzte das von Stricker verwendete Bienenwachs und Tristearin durch Paraffin und führte Letzteres auf diesem Wege in die Histologie ein. Er träufelte geschmolzenes Paraffin auf das Gewebe, das zuvor mit oder ohne Alkohol vorbereitet worden war [18, S. 207–208 Fußnote]. Wie er feststellte, waren die Schnitte besser als die von frischem Gewebe [18, S. 215].

In einem späteren Beitrag schrieb Klebs, dass er Paraffin seit 5 Jahren verwendete und dass andere Wissenschaftler, einschließlich Wilhelm His Senior, es ebenfalls für nützlich hielten [19, S. 164]. Er monierte aber auch, dass das Wachs nicht vollständig am Gewebe anhaftete, sodass Hohlräume bestanden. Diese Hohlräume sind störend, da sie dem Gewebe beim Schneiden Bewegungsspielraum bieten [19, S. 165]. Die geringe Mischbarkeit von Paraffin mit Alkohol ist eine wahrscheinliche Erklärung für diese Probleme. Jedenfalls rückte Klebs vom Paraffin ab und wendete sich stattdessen einer Mischung von Glycerin und Hausenblase (Fischleim) zu, die in das Gewebe eindringt [19, S. 165].

Seltsamerweise wird Klebs’ Veröffentlichung von 1869 häufig zitiert, um die Einführung von Paraffin in die Histologie zeitlich zu bestimmen [6, 40], wo sie doch eigentlich Klebs’ Abkehr von Paraffin markiert. Dieses Missverständnis könnte seinen Ursprung in Longs 1928 erschienenem Buch *A History of Pathology* genommen haben (Nachdruck in [25]).

### Wilhelm His Senior (1868) – Infiltrationseinbettung mit Paraffin

**Figure 1:**
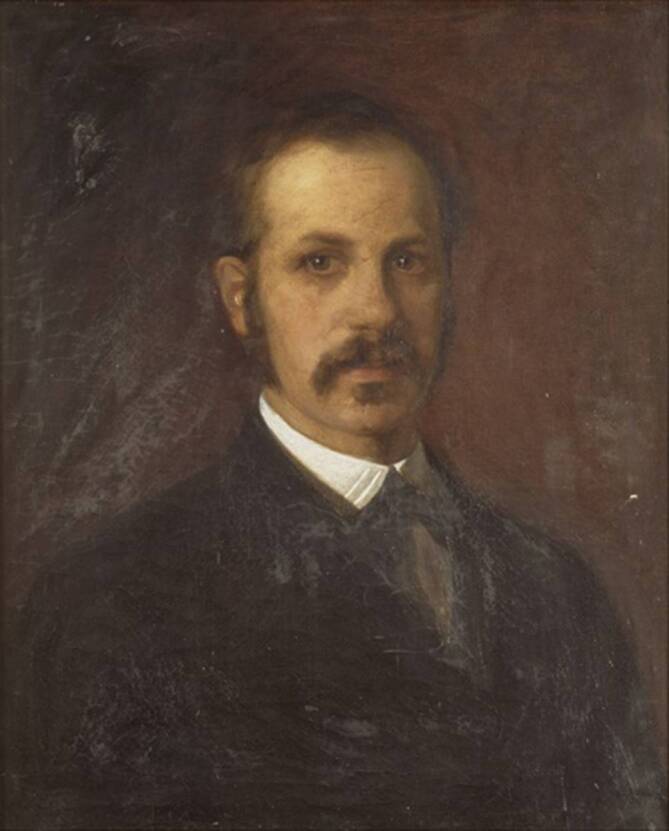

Wilhelm His Sen. (1831–1904) war Embryologe und Professor für Anatomie und Physiologie, zunächst an der Universität Basel, später in Leipzig (Abb. 1). Er publizierte eine Vielzahl bedeutender Forschungsarbeiten im Bereich der Pathologie, Anatomie und Embryologie [8]. Sein Sohn Wilhelm His Junior (1863–1934) ist der Entdecker des Fasciculus atrioventricularis (His-Bündel) [1].

His Senior hielt es für unmöglich, mit den Methoden seiner Zeit qualitativ hochwertige Schnitte anzufertigen [15]. Als er erfuhr, dass Klebs Paraffin verwendete, erkannte er dessen Potenzial [15, S. 181]. In der Folge wandelte er Klebs’ Verfahren ab, indem er es um eine Entwässerung mit Alkohol und eine Klärung mit Lavendelöl oder Kanadabalsam ergänzte. Als Klärmittel verwendete er Lavendelöl (destilliert aus *Lavandula* sp., häufig *L. angustifolia*).

Wilhelm His hielt an Paraffin fest, wo Klebs davon abgekommen war, wie Klebs selbst kommentierte [19, S. 164]. His bezeichnete Paraffin als „vorzüglichen Stoff“, von dem er durch Klebs erfahren habe [15, S. 181]. Dass His Lavendelöl verwendete, ist ausgesprochen interessant, da es als Intermedium gewirkt und das Eindringen des Wachses in das Gewebe ermöglicht haben könnte. Somit könnte His, vielleicht zufällig, den Prozess der Infiltrationseinbettung mit Paraffin entdeckt haben.

**Figure 2:**
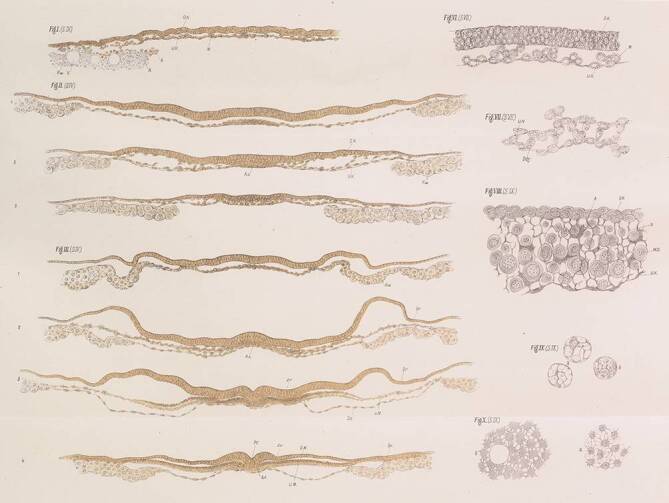

His beschrieb sein Protokoll für die Einbettung von Hühnerembryonen in der Monografie zur Entwicklung des Hühnchens im Ei [15, S. 180–182; Nachdruck im Rahmen des vorliegenden Beitrags, Zusatzanmerkung 1]. Das Protokoll scheint Schnitte von hoher Qualität ermöglicht zu haben (Abb. 2). His entwässerte Embryonen mithilfe einer aufsteigenden Alkoholreihe und tränkte sie in Lavendelöl als Klärmittel, um sie für eine Untersuchung im Ganzen transparent zu machen. Gelegentlich legte er einen Embryo in Kanadabalsam auf und schloss ihn mit Deckgläsern in einem gekammerten Objektträger ein, sodass er ihn von beiden Seiten betrachten konnte (Entwässerung und Klärung waren bereits etablierte Verfahren [43, S. 12]).

In Lavendelöl geklärte Embryonen wurden daraufhin eingebettet. His legte die Embryonen auf ein Guttaperchaplättchen und träufelte Paraffin darüber. Nach Anfertigung von Schnitten mit seinem eigenen Mikrotom [13] legte er diese auf Glasträger und entfernte das Paraffin mit Chloroform oder Benzin [15, S. 181].

Hensen erinnerte sich, dass His die Verwendung von Paraffin befürwortete [11]. Apáthy spricht von der „Paraffinmethode von His“ [2, S. 83]. Nur ein Jahr nach His’ Publikation wendete Dr. Moritz Roth in Greifswald die „von W. His für Embryonen angegebene Methode“ an [34, S. 246]. Waldeyer schreibt die Paraffineinbettung Klebs und His zu [42].

So wie die Paraffineinbettung heutzutage von Pathologen und Forschern durchgeführt wird, gleicht sie in den grundlegenden Schritten bemerkenswert der Technik von His. Die wichtigsten Verbesserungen betreffen die Wahl der Zwischenflüssigkeit, die Rezeptur der Paraffinmischung und das Schnittverfahren, für das heutzutage automatische Mikrotome verwendet werden, die routinemäßig Schnitte von 5–7 μm liefern. Für Details zur Verbesserung der Paraffineinbettung im Laufe der Jahre siehe [22, 31].

### Protokoll von His und dessen Überprüfung

Betrachtet man die Qualität der in His’ Monografie dargestellten Schnitte, ist ihm vermutlich eine Infiltration mit Paraffin gelungen. Im vorliegenden Beitrag gehen wir der Frage nach, ob mit dem von ihm veröffentlichten Protokoll [15] tatsächlich eine Infiltration erreicht werden konnte.

Wir versuchten, das Protokoll von His detailgetreu zu befolgen. His berichtete, dass er heißes Wachs auf die Gewebe träufelte, während diese auf einem Guttaperchaplättchen lagen. Allerdings geht er kaum ins Detail. Nach unserer Vermutung verwendete His Guttapercha, weil dessen geringe Wärmeleitfähigkeit [28] dafür sorgte, dass das Wachs eine gewisse Zeit geschmolzen blieb, bevor es abkühlte und fest wurde. Da wir kein Guttaperchaplättchen beschaffen konnten, verwendeten wir Plättchen aus Bakelit oder Kork, die beide eine geringe Wärmeleitfähigkeit haben [10, 23]. Da es uns nach dem Protokoll von His nicht gelang, brauchbare Schnitte herzustellen, testeten wir verschiedene Modifikationen (Zusammenfassung in Tab. 1). Unter anderem nahmen wir einen zusätzlichen Verfahrensschritt mit Einschmelzen der in Paraffin eingebetteten Embryonen in frisch geschmolzenem Paraffin auf. In allen Fällen fertigten wir 50-µm-Schnitte an, da dies die von His verwendete Standarddicke für das Schneiden von Hühnerembryonen war [14, S. 383].

**Table 1:** 

*N*	Fix	Entwässerung Protokoll	50:50	Intermedium	Abtupfen	Basis	Trocknung	Beträufeln	Erneute Einbettung	Ergebnis
5	OsO_4_	50 % (1 h), 70 % (1 h), 2‑mal 100 % (jeweils 1 h)	Nein	Lavendel	Nein	Bakelit	Nein	Ja	Nein	^a^
1	OsO_4_	50 % (1 h), 70 % (1 h), 2‑mal 100 % (jeweils 1 h)	Ja	Lavendel	Nein	Bakelit	Nein	Nein	Nein	^b^
1	OsO_4_	50 % (1 h), 70 % (1 h), 2‑mal 100 % (jeweils 1 h)	Nein	Lavendel	Ja	Bakelit	Nein	Ja	Nein	^a^
1	OsO_4_	50 % (1 h), 70 % (1 h), 2‑mal 100 % (jeweils 1 h)	Nein	Lavendel	Ja	Bakelit	Nein	Ja	Ja	^d^
1	OsO_4_	50 % (1 h), 70 % (1 h), 2‑mal 100 % (jeweils 1 h)	Nein	Lavendel	Ja	Bakelit	Nein	Ja	Nein	^a^
1	OsO_4_	50 % (1 h), 70 % (1 h), 2‑mal 100 % (jeweils 1 h)	Nein	Lavendel	Ja	Bakelit	30 min	Ja	Nein	^a^
1	OsO_4_	50 % (1 h), 70 % (1 h), 2‑mal 100 % (jeweils 1 h)	Nein	Lavendel	Ja	Bakelit	1 h	Ja	Nein	^a^
2	OsO_4_	50 % (1 h), 70 % (1 h), 2‑mal 100 % (jeweils 1 h)	Nein	Lavendel	Ja	Kork	Nein	Ja	Nein	^a^
1	OsO_4_	50 % (1 h), 70 % (1 h), 2‑mal 100 % (jeweils 1 h)	Nein	Lavendel	Ja	Kork	30 min	Ja	Nein	*
1	OsO_4_	50 % (1 h), 70 % (1 h), 2‑mal 100 % (jeweils 1 h)	Nein	Lavendel	Ja	Kork	1 h	Ja	Nein	^a^
2	OsO_4_	50 % (1 h), 70 % (1 h), 2‑mal 100 % (jeweils 1 h)	Nein	Lavendel	Ja	Kork	Nein	Ja	Ja	^d^
2	OsO_4_	50 % (1 h), 70 % (1 h), 2‑mal 100 % (jeweils 1 h)	Nein	Lavendel	Ja	Bakelit	Nein	Ja	Nein	^a^
2	OsO_4_	50 % (1 h), 70 % (1 h), 2‑mal 100 % (jeweils 1 h)	Nein	Lavendel	Ja	Bakelit	Nein	Ja	Ja	^e^
2	OsO_4_	50 % (1 h), 70 % (1 h), 2‑mal 100 % (jeweils 1 h)	Ja	Lavendel	Nein	Kunststoff	Nein	Nein	n.a.	^b,c^
6	Bouin	50 % (1 h), 70 % (1 h), 95 % (1 h), 3‑mal 100 % (jeweils 1 h)	Ja	Histo-Clear	Nein	n.a.	Nein	Nein	Nein	^e^

*N* Embryonenzahl, *Fix* Fixiermittel, *50:50* Mischung gleicher Anteile von Intermedium und Paraffin vor Einbettung in reinem Paraffin, *Abtupfen* Abtupfen von Lavendelöl vor Zugabe von Paraffin, *Beträufeln* Beträufeln des Embryos mit Paraffin, *n.a.* nicht anwendbar

Spalte „Ergebnis“: ^a–e^zeigen die subjektiv eingeschätzte Schnittqualität an: ^a^schlechte Qualität, Gewebe stark zerrissen, ^e^hervorragende Qualität, keine Risse

## Material und Methoden

### Erklärung zur Einhaltung ethischer Richtlinien

Alle tierexperimentellen Verfahren wurden im Einklang mit lokalen und internationalen Bestimmungen durchgeführt. Die lokalen Bestimmungen werden geregelt durch das *Wet op de dierproeven* („Tierversuchsgesetz“; Artikel 9) des niederländischen Rechts (national), das vom Büro für die Genehmigung von Tierversuchen der Universität Leiden (lokal) verwaltet wird. Mit dieser lokalen Regelung ist die *Richtlinie zum Schutz von Versuchstieren* (Rat der Europäischen Union, Richtlinie 86/609/EEC) umgesetzt, welche die Verwendung von Hühnerembryonen vor dem Zeitpunkt des Schlüpfens erlaubt (ungefähr 21 Tage der Inkubation bei 38 °C). Da die hier verwendeten Embryonen nicht länger als 3 Tage inkubiert wurden, war keine Genehmigung nach der Richtlinie 86/609/EEC des Europarats (1986) oder von der Ethikkommission der Universität Leiden erforderlich.

### Embryonen

Befruchtete Eier des Weißen Leghorn-Huhns (*Gallus gallus*) wurden von einem gewerblichen Anbieter bezogen (Drost Loosdrecht B. V, Loosdrecht, Niederlande). Die Eier wurden für 2,5 Tage bei 38 °C in einem Inkubator mit Luftbefeuchter und unbeweglichen Ablagen inkubiert. Das Entwicklungsstadium der Embryonen wurde gemäß der Klassifikation von Hamburger und Hamilton bestimmt, dann wurden sie aus den Eiern in phosphatgepufferte Salzlösung (PBS) verbracht.

### Fixierung, Einbettung und Schnittführung

**Figure 3:**
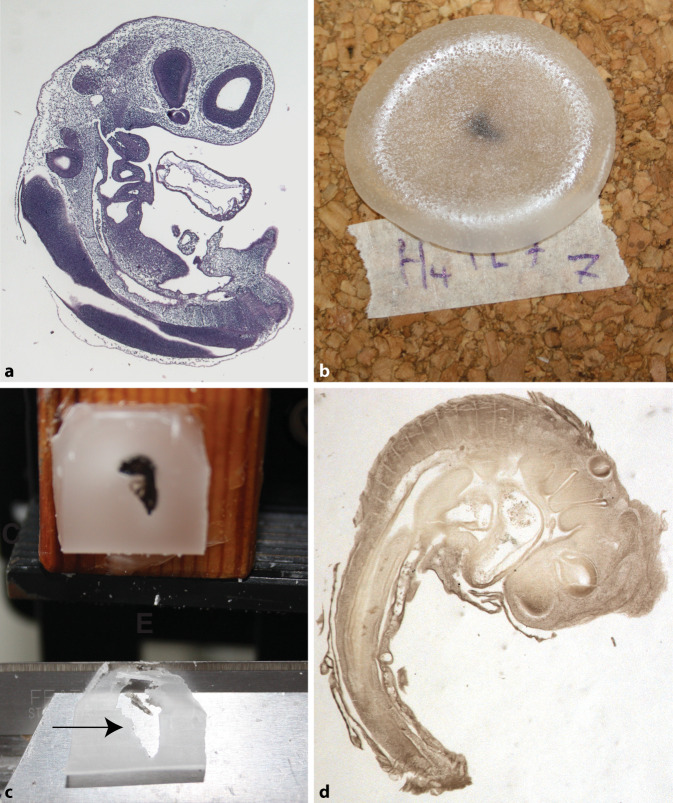

Als Positivkontrollen wendeten wir zunächst herkömmliche histologische Präparationsverfahren an [4, 26, 32], um Schnitte von 2,5 Tage alten Hühnerembryonen anzufertigen (7 µm, Hämatoxylin-Eosin-Färbung; Abb. 3a). Daraufhin versuchten wir, Schnitte von 2,5 Tage alten Hühnerembryonen nach dem von His beschriebenen Protokoll (Zusatzanmerkung 1) herzustellen. Da im Protokoll Details fehlten, nahmen wir einige Änderungen vor (aufgeführt in Tab. 1). Kurz zusammengefasst wurden die Embryonen durch Beträufeln mit 0,5 % Osmium(VIII)-oxid-Lösung fixiert bis eine Braunfärbung einsetzte (30–60 s). Danach wurden sie in einer aufsteigenden Ethanolreihe bis 100 % entwässert und über Nacht mit einem Intermedium geklärt, entweder Lavendelöl (*Lavendula officinalis*; www.berivita.com) oder Histo-Clear™ (National Diagnostics, Atlanta, USA).

Mehrere Embryonen wurden bei 62 °C durch Beträufeln mit geschmolzenem Paraffin (Paraclear; Klinipath, Duiven, Niederlande) eingebettet (Abb. 3b), wie von His beschrieben. Dafür wurden die Embryonen aus dem Lavendelöl auf ein Bakelit- oder Korkplättchen überführt, wobei ungefähr 20 µl Lavendelöl an ihnen haftete. Nun wurden etwa 2,5 g geschmolzenes Paraffin (62 °C) darüber geträufelt. In einigen Fällen wurde zunächst das überschüssige Lavendelöl mit Filterpapier entfernt oder trocknen gelassen, entweder bis das restliche Öl verdunstet oder bis vollständige Trockenheit erreicht war (Tab. 1). Folgende weitere Varianten gab es: Einige Embryonen wurden aus dem Lavendelöl in eine 50:50-Mischung von Lavendelöl und Paraffin überführt (62 °C, 1 h) und dann in geschmolzenes Paraffin eingebettet. Andere wurden – nach Behandlung gemäß dem Protokoll von His, Beträufelung mit Paraffin und Abkühlen – in frisch geschmolzenem Paraffin eingeschmolzen und eingebettet.

In allen Fällen wurden die in Paraffin eingebetteten Embryonen zur weiteren Verfestigung über Nacht belassen, mit einer Rasierklinge vom Plättchen gelöst und für die Anfertigung von 50-µm-Schnitten auf 3 cm^3^ großen Kiefernholzblöckchen befestigt. Die Schnitte wurden entwachst und mit Eukitt (Sigma-Aldrich, jetzt Merck KGaA, Darmstadt, Deutschland) eingedeckt. Die in Histo-Clear (letzte Reihe in Tab. 1) verarbeiteten Embryos wurden mit Hämatoxylin und Eosin gefärbt.

## Ergebnisse

Wir verwendeten Kork- oder Bakelitplättchen als Basis (Guttapercha war nicht erhältlich). Auf diesen Substraten blieb das Wachs über eine beträchtliche Zeitspanne flüssig (25 min bzw. 7 min), dennoch erhielten wir keine brauchbaren Schnitte (Abb. 3c). Wurde geschmolzenes Paraffin auf Embryonen geträufelt, an denen etwa 20 µl Lavendelöl haftete, drang das Paraffin nicht in das Gewebe. Die Schnitte zerfielen, wenn sie auf die Objektträger verbracht wurden, wahrscheinlich wegen der Persistenz von Lavendelöl im Einbettprozess. Nur mit einigen wenigen verbliebenen Gewebestreifen gelangen zufriedenstellende Schnitte. Das Abtupfen von überschüssigem Lavendelöl bewirkte eine mäßige Verbesserung bei manchen Schnitten, führte aber ebenfalls nicht zu annehmbaren Schnittergebnissen. Wir vermuteten, dass das Paraffin nicht lange genug geschmolzen blieb, um sich mit dem Lavendelöl zu mischen und in das Gewebe einzudringen. Zur Prüfung dieser Hypothese ergänzten wir das His-Protokoll um einen zusätzlichen Schritt: Die Probe (die mit Paraffin beträufelt worden war) wurde erneut geschmolzen und dann in geschmolzenes Paraffin gelegt (bei 62 °C im Ofen). Das Ergebnis war eine enorme Verbesserung der Paraffininfiltration und eine entsprechende Verbesserung der Schnittqualität (Abb. 3c). Eine alternative Ergänzung von His’ Protokoll wurde ebenfalls getestet: ein Infiltrationsschritt in einer 50:50-Mischung von Lavendelöl und Paraffin. Dies führte zu einer gewissen Verbesserung der Schnittqualität, aber in einem geringeren Maße als das Verfahren mit erneutem Schmelzen und verlängerter Infiltration in geschmolzenem Paraffin.

## Diskussion

Mehr als früher wird jetzt von Demjenigen, welcher eine Untersuchung mittheilt, verlangt, dass er auch die angewendeten Untersuchungsmethoden einlässlich darlege. Dieser Forderung will ich in den nachfolgenden Zeilen zu genügen suchen (Wilhelm His Sen. [15]).

Es erscheint uns wenig wahrscheinlich, dass mit dem von His 1868 beschriebenen Protokoll [15] Schnitte von ausreichender Qualität angefertigt werden können. Sicherlich nicht die hervorragenden Schnitte, die in seiner Arbeit dargestellt sind (Abb. 2). Ebenso wenig die lückenlose Serie qualitativ hochwertiger Schnitte, die er gebraucht haben muss, um seine herrlichen 3D-Modelle von Embryonen herzustellen [16]. Wenn wir sein Protokoll streng befolgten, war das Gewebe schlecht infiltriert und die Schnitte größtenteils zerrissen und unbrauchbar. Nur bei Ergänzung des His-Protokolls um einen Infiltrationsschritt mit geschmolzenem Paraffin hatten die Schnitte eine annehmbare Qualität.

Möglicherweise haben wir His’ Protokoll nicht genau genug befolgt, auch wenn mehrere Varianten seines Verfahrens erfolglos getestet wurden. Selbst wenn wir die Embryonen 25 min in heißem Wachs beließen (durch Beträufeln der auf einem Korkplättchen ruhenden Proben mit geschmolzenem Paraffin), wurde keine Paraffininfiltration erreicht. Denkbar ist, dass sich die von uns und von His verwendeten Paraffine in ihren Eigenschaften unterschieden. Wir verwendeten Paraclear, das aus Paraffin und einigen zugesetzten Kunststoffpolymeren besteht (nach Angaben des Herstellers Sigma Merk ist die genaue Zusammensetzung ein Betriebsgeheimnis).

Das wirft die Möglichkeit auf, dass His eine längere Infiltration durchführte, dies aber nicht in seinem Protokoll vermerkte. Eine andere mögliche Erklärung ist, dass His Wachs mit einer viel höheren Temperatur verwendete als die 62 °C, die heute in histologischen Standarduntersuchungen üblich sind (und auch von uns gewählt wurden). Prinzipiell könnte sehr heißes Wachs länger flüssig geblieben und schneller ins Gewebe eingedrungen sein. Leider gibt His die Temperatur des von ihm verwendeten Wachses nicht an.

Die Lücken in His’ Protokoll könnten ein simples Versehen sein. Ansonsten wollte er vielleicht wissenschaftliche Konkurrenten daran hindern, seine Technik nachzuahmen. Erwähnenswert ist auch, dass die Anfertigung von Schnitten die Grundlage seiner kommerziell erfolgreichen Modelle war. Wir sind der Ansicht, dass His für die bahnbrechende Erfindung der Infiltrationseinbettung mit Paraffin gewürdigt werden sollte. Es ist bedauerlich, dass His kein vollständiges Protokoll veröffentlicht hat, denn ein solches hätte seine wichtige Innovation für die Nachwelt festgehalten.

## Supplementary Information



## References

[1] Anderson RH, Mori S (2016). Wilhelm His junior and His bundle. J Electrocardiol.

[2] Apáthy S (1896). Die Mikrotechnik der tierischen Morphologie: Eine kritische Darstellung der mikroskopischen Untersuchungsmethoden.

[3] Arko-Boham B, Ahenkorah J, Hottor BA (2014). Improved method of producing satisfactory sections of whole eyeball by routine histology. Microsc Res Tech.

[4] Baker JR (1975). Cytological technique.

[5] Bird CHG (1875). Imbedding in elder pith for cutting sections. Q J Microsc Sci.

[6] Bonsett CA, Rudman A (1994). ‘Oil globules’ in Duchenne muscular dystrophy—history, demonstration, and metabolic significance. Med Hypotheses.

[7] Fearnley W (1887). A course of elementary practical histology.

[8] Fick R (1904). Wilhelm His. Anat Anz.

[9] Foster M (1870). On imbedding substances for microscopic section. Q J Microsc Sci.

[10] Gil L (2015). New cork-based materials and applications. Materials.

[11] Hensen V (1876). Beobachtungen über die Befruchtung und Entwicklung des Kaninchens und Meerschweinchens. Z Anat Entwicklungsgesch.

[12] Hill J (1770) The construction of timber from its early growth (etc.). Self-published, London

[13] His W (1870). Beschreibung eines Mikrotoms. Arch Mikrosk Anat.

[14] His W (1887). Über die Methoden der plastischen Rekonstruktion und über deren Bedeutung für Anatomie und Entwickelungsgeschichte. Anat Anz.

[15] His W (1868). Untersuchungen über die erste Anlage des Wirbelthierleibes. Die erste Entwickelung des Hühnchens im Ei.

[16] Hopwood N (2002). Embryos in wax; models from the Ziegler studio. Whipple museum of the history of science.

[17] Kanitz A (1880). Eduard Fenzl. Eine Lebensskizze. Bot Z.

[18] Klebs (1867). Bemerkungen über Larynx-Geschwülste. Arch Pathol Anat Physiol Klin Med.

[19] Klebs E (1869). Die Einschmelzungs-Methode, ein Beitrag zur mikroskopischen Technik. Arch Mikrosk Anat.

[20] Klebs E (1876). Eine Schneidemaschine zur Anfertigung mikroskopischer Präparate, nebst Bemerkungen über mikroskopisches Schneiden. Archiv f. experiment. Pathol. u. Pharmakol.

[21] Lee AB (1885). The microtomist’s vade-mecum; a handbook of the methods of microscopic anatomy.

[22] Lee AB, Mayer P (1901). Grundzüge der mikroskopischen technik für zoologen und anatomen.

[23] Lepetit J, Favier R, Grajales A (2004). A simple cryogenic holder for tensile testing of soft biological tissues. J Biomech.

[24] Lewis F, Maughan NJ, Smith V (2001). Unlocking the archive—gene expression in paraffin-embedded tissue. J Pathol.

[25] Long ER (1965). A history of pathology.

[26] Mcauliffe WG (2013). Routine histology techniques for the developing and adult central nervous system. Methods Mol Biol.

[27] Miller DV, Jensen TA, Bair TL (2020). A novel, rapid, and low cost method for preparing tissues with metallic stents for routine histology. Cardiovasc Pathol.

[28] Miner MR, Berzins DW, Bahcall JK (2006). A comparison of thermal properties between gutta-percha and a synthetic polymer based root canal filling material (Resilon). J Endod.

[29] Mosse M, Ehrlich P (1910). Enzyklopädie der mikroskopischen Technik (A-K).

[30] Mosse M, Ehrlich P (1910). Enzyklopädie der mikroskopischen Technik (L-Z).

[31] Mulisch M, Welsch UE (2015). Romeis: Mikroskopische Technik.

[32] Nation B, Orchard G (2012). Histopathology.

[33] Pereira J (1842). The elements of materia medica and therapeutics.

[34] Roth M (1869). Zur Frage von der Bindesubstanz in der Grosshirnrinde. Arch Pathol Anat Physiol Klin Med.

[35] Schacht H (1862). Das Mikroskop und Seine Anwendung, Insbesondere für Pflanzen-Anatomie.

[36] Smith GM (1915). The development of botanical microtechnique. Trans Am Microsc Soc.

[37] Sperber O, Kaminsky W, Geißler A (2005). Structure analysis of paraffin waxes by 13C-NMR spectroscopy. Petroleum Sci Technol.

[38] Stilling B, Wallach J (1842). Untersuchungen über die Textur des Rückenmarks.

[39] Stricker S (1864). Untersuchungen über die Entwickelung des Kopfes der Batrachier. Arch Anat Physiol Wiss Med.

[40] Van Den Tweel JG, Taylor CR (2010). A brief history of pathology: Preface to a forthcoming series that highlights milestones in the evolution of pathology as a discipline. Virchows Arch.

[41] Van Langevelde A, Peschar R, Schenk H (2001). Structure of beta-trimyristin and beta-tristearin from high-resolution X-ray powder diffraction data. Acta Crystallogr B.

[42] Waldeyer W (1891). Ueber einige neuere Forschungen im Gebiete der Anatomie des Centralnervensystems.

[43] Welcker H (1856). Ueber Aufbewahrung mikroskopischer 0bjecte nebst Mittheilungen über das Mikroskop und dessen Zubehör.

