# Supplementary material for: Wilhelm His, Sr., and the development of paraffin embedding. German version
Source: Pathologe. 2021 May 13;42(4):424–30. [Article in German] doi: 10.1007/s00292-021-00943-8 (PMC8249263; doi:10.1007/s00292-021-00943-8)
Supplement: Supplementary file 1 [file 292_2021_943_MOESM1_ESM.docx]

# SUPPLEMENTARY INFORMATION zu

**Wilhelm His senior und die Entwicklung der Paraffineinbettung**

Tim van der Lem1; Merijn de Bakker1; Gerhard Keuck2; Michael K. Richardson1,*

1) Institute of Biology, Leiden University (IBL), Sylvius Laboratory, Sylviusweg 72, 2333 BE, Leiden, Niederlande

2) Geißspitzweg 8, 65929 Frankfurt, Deutschland. Ehemals Bayer CropScience, Frankfurt, und Hoechst AG, Deutschland.

*Korrespondierender Autor, E-Mail: m.k.richardson@biology.leidenuniv.nl

**Das histologische Verfahren von His** (ursprünglicher deutscher Text). Aus [15] S. 180-182, Originalfassung mit Fußnoten.

**III. Methoden der Untersuchung.**

Die Behandlung sehr junger Keime gehört zu den Dingen, bei deren Erlernung wohl jeder einiges Lehrgeld wird zu bezahlen haben. Wie weit man aber bei geschickter Hand und bei der nöthigen Ausdauer schon mit den allereinfachsten Hülfsmitteln gelangen kann, das zeigen jene bewundernswürdigen Arbeiten der Begründer der Embryologie, C. Fr. Wolff's, Döllinger's und Pander's, v. Baer's, Rathke's u. A., Arbeiten, deren Ergebnissen Neues beizufügen, auch einer weit complicirteren Technik nur mühsam gelingt.

Einige Notizen über die Präparationsweise der Würzburger Schule finden sich in der lateinischen Dissertation Pander's. Aus ihnen geht hervor, dass er, und seine Mitarbeiter die Eier stets unter Wasser eröffnet haben, ein Verfahren, das, der Döllinger‘schen Tradition folgend, späterhin auch Erdl empfohlen hat, und das in neuester Zeit wiederum von Moleschott und von Dursy angenommen worden ist. Etwas Maceration in Wasser und Auseinanderziehen mit Nadeln sind die einzigen weiteren Kunstgriffe, welche von den älteren Beobachtern scheinen angewendet worden zu sein.

Mehr als früher wird jetzt von Demjenigen, welcher eine Untersuchung mittheilt, verlangt, dass er auch die angewendeten Untersuchungsmethoden einlässlich darlege. Dieser Forderung will ich in den nachfolgenden Zeilen zu genügen suchen, und ich bemerke dabei, dass mir im Allgemeinen die von mir befolgten Methoden der Ei-Manipulation gute Dienste geleistet haben, dass aber sicherlich auch andere Wege ebenso gut, und für specielle Zwecke selbst besser zum Ziel führen können.

Das Ei habe ich jeweilen nach dem, erst kürzlich (durch v. Baer) mitgetheilten Würzburger Kunstgriff am stumpfen Pol eingeschlagen, die Schaale aufgebrochen, und nun den Inhalt in ein flaches Glasgefäss ausgegossen^[[1]](#footnote-1)^. Schon vor dem Ausgiessen, oder gleich nachher werden die Chalazen dicht am Dotter durchschnitten, was zur Folge hat, dass nun die innerste zähe Eiweisslage als zusammenhängende Hülse von der Dotterhaut sich abziehen lässt. Der Keim wird durch geeignete Drehung des Dotters zu oberst gebracht, kreisförmig umschnitten, und durch ein angelegtes grosses Deckglas vom übrigen Dotter abgehoben. Diese Abhebung misslingt nur dann, wenn zuvor das Eiweiss von der Dotterhaut nicht gehörig entfernt war. Bei Embryonen vom 2. Tage erfolgt die Abhebung bei gehöriger Vorsicht so reinlich, dass der Embryo ohne Weiteres unter das Mikroscop gebracht werden kann. Bei jüngeren Keimen aber pflegt dem abgehobenen Präparat eine kleinere oder grössere Dottermenge zu folgen. Bei unbebrüteten Keimscheiben löst sich meist der ganze Boden der Keimhöhle nebst etwas gelbem Dotter mit aus.

Die Reinigung des abgelösten Keimes von anhängendem Dotter und die Entfernung der Dotterhaut wird durch Jodserum vorgenommen, das ich mittelst einer Pipette in sehr sanftem Strahl über das Präparat leite. Bei dieser und den folgenden Operationen bis zur Durchschneidung wird das Deckglas stets als Unterlage und geeignetes Transportmittel des Präparates beibehalten, das unter diesen Umständen möglichst wenig Verzerrungen und Faltungen erleidet^[[2]](#footnote-2)^.

Den gereinigten, seinem Deckglas anhaftenden Keim pflege ich nun unmittelbar unter das Mikroscop zu bringen. Man kann ihn bei schwächeren und mittleren Vergrösserungen leicht von der Rücken- sowohl, als von der Bauchseite beobachten, wenn man sich jener einfachen, von Kühne angegebenen feuchten Kammer bedient, die in einem durchbohrten, unten mit einer Platte verschlossenen Objecträger besteht. Natürlich muss die Oeffnung gross genug sein, damit bei abwärts gerichtetem Keim ihre Ränder nirgends den letzteren berühren.

Nach Beendigung der Vorstudien am frischen Keim, setze ich demselben einige Tropfen von Überosmiumsäurelösung (0,5%) zu, bis zum Eintritte leicht bräunlicher Färbung. Hierauf wird die Säure mit schwachem Weingeist abgespült, der Keim erst in schwächeren, dann in stärkeren und endlich in absoluten Alkohol gelegt, und nach genügender Entwässerung in ätherisches Oel (Lavendelöl)^[[3]](#footnote-3)^.

Also vorbereitet kann der Keim zwischen zwei Deckgläser in Canadabalsam eingeschlossen, und auf durchbohrtem Objectträger festgekittet werden. Diese Befestigung hat den Vortheil, dass das Präparat beiderseits die Beobachtung mit starken Vergrösserungen zulässt. — Soll der Keim zu Schnitten verwendet werden, so pflege ich ihn erst nochmals unter das Mikroskop zu bringen, und mit der Camera lucida bei schwächerer Vergrösserung (Hartnak, System I oder II) zu zeichnen, dann schliesse ich ihn in Paraffin ein, das einem Guttaperchaplättchen aufgetropft wird. Die Verwendung dieses vorzüglichen Stoffes zu mikroskopischen Zwecken halte ich durch Klebs zuerst kennen gelernt. Das Zerlegen des Keimes geschieht mittelst eines besonderen, an einem anderen Ort ausführlich zu schildernden Apparates. — Das Princip des Schneidapparates beruht darauf, dass das Object mittelst einer Mikrometerschraube unter einer vertical stehenden Stahlplatte durchgeführt wird, längs deren das, auf einer Seite flach geschliffene Rasirmesser gleitet. Die erste Veranlassung zur Herstellung des Apparates gab mir der Hensen'sche Querschnitter. Noch leichter, wie mir scheint, als dieser erlaubt er die successive Zerlegung des ganzen Keimes in beliebig gerichtete, und beliebig feine Schnitte, ohne irgend welchen Verlust. Die Schnitte werden einzeln auf den Objectträger gebracht, mit Chloroform, oder Benzin vom anhängenden Paraffin gereinigt, und in Canadabalsam eingekittet. Die eingekitteten Schnitte, nach ihrer Reihenfolge numerirt, können nun natürlich nicht nur unter einander, sondern auch mit der Zeichnung des unverletzten Embryo auf das Genaueste verglichen werden.

Die ganze Operation von Eröffnung des Eies bis zur Zerlegung in Schnitte lässt sich in 12—24 Stunden durchfuhren. In der Regel zerschneide ich an einem Tage die, am Tage zuvor aus dem Ei genommenen Embryonen, und ich habe mich stets weniger gut dabei befunden, wenn die Sache sich mehr in die Länge zog. Bei längerem Aufenthalt in Alkohol, oder in ätherischen Oelen tritt nämlich eine Schrumpfung der Theile ein, welche bei abgekürztem Verfahren nicht in merklichem Maasse sich einstellt.

Auf die Herstellung möglichst vollständiger Schnittserien lege ich nicht minder, als auf die vorangehende Entwerfung einer exacten Zeichnung des unzerschnittenen Embryo grosses Gewicht. Schon bei jüngeren Keimen, von denen kaum einer dem anderen gleicht, ist ein sicheres Verständnis nur durch möglichst allseitige Vergleichung aller Verhältnisse eines und desselben Objectes erreichbar, allein vollends bei älteren vom 2., 3. und 4. Tage lässt die Betrachtung mancher Durchschnitte in vielen Fällen völlig rathlos, wenn wir nicht auf das Genaueste die Lage des Schnittes und seine Beziehungen zu den vorhergehenden und nachfolgenden kennen.

Das Ideal der Untersuchung wäre das, dass wir denselben Embryo erst von der Fläche betrachten, und dann in Längs- und in Querschnitten untersuchen könnten, so dass wir, ähnlich den Architecten, zum gegebenen Grundriss die verschiedenen Aufrisse erhielten. Dies Ideal kann man zwar nicht direct erreichen, wohl aber auf einem Umweg, und dieser ist, wenigstens für die Beurtheilung etwas älterer Embryonen (vom 2 bis 4 Tage), werthvoll genug. — Hat man eine Flächenansicht und die zugehörige Serie von Querschnitten mit der Camera lucida gezeichnet, und man kennt genau die Stellung jedes Querschnittes zur Axe, so kann man sich die Längsaxe des Rückens in annähernd richtiger Krümmung auf ein Papier zeichnen, mit dem Zirkel je die verschiedenen Tiefenabstände des Medullarrohres , der Aorta und so weiter an den Querschnitten abmessen, und am richtigen Ort eintragen. Man erhält so einen Sagittalschnitt, der für ältere Embryonen von dem wirklich führbaren Schnitt dadurch sich unterscheidet, dass alle Punkte, die der gekrümmten Medianfläche angehören, in eine Ebene zu liegen kommen, als ob der Embryo zurückgekrümmt, und seine Medianfläche in eine Ebene ausgebreitet worden wäre. In ähnlicher Weise kann man natürlich auch Frontalschnitte , oder beliebig anders gerichtete Schnitte construiren.

Mit Hülfe der verschiedenen Durchschnitte eines und desselben Präparates lassen sich nun auch körperliche Modelle desselben anfertigen. — Ich habe mir in einem früheren Stadium meiner Untersuchung aus biegsamem Material, aus Bleiblech und Leder, eine Anzahl von Modellen gemacht, und bei deren Herstellung Mancherlei gelernt. Die Consequenzen des Faltungsprincipes drängen sich bei derartigen Arbeiten mit einer viel unwiderstehlicheren Gewalt auf, als bei der blosen Betrachtung von Schnitten. — Immerhin sind die fraglichen Materialien etwas unfügsam, sobald es sich um complicirtere Formungen handelt, und ich bin auch bald auf dem Punkte angelangt, wo sie mich im Stiche gelassen haben. Hier würde ich vielleicht stehen geblieben sein, hätte ich nicht in dem bewährten Freiburger Künstler Herrn Dr. A. Ziegler eine kräftige Hülfe gefunden. Durch ihn wurde ich mit der Handhabung von Modellirthon und von Wachs vertraut gemacht, und in gemeinschaftlicher Arbeit haben wir nach Präparaten und Durchschnitten eine Serie von Modellen hergestellt, bei deren Anfertigung möglichste Genauigkeit und Naturtreue angestrebt wurde. Wer eine derartige plastische Arbeit nicht versucht, macht sich kaum einen Begriff von der scharfen Controlle, die dieselbe gewährt. Jede Einzelheit, jede scheinbare Unregelmässigkeit eines Schnittes erhält dabei ihre eigenthümliche Bedeutung, jede Unschärfe rächt sich durch einen Fehler im plastischen Objecte, und die detaillirte Durcharbeitung solcher Modelle mit Zirkel und Maasstab giebt schliesslich eine Sicherheit der Anschauung, wie sie wohl auf anderem Wege kaum erreichbar ist.

1. Obige Methode, das Ei zu eröffnen, ist weit einfacherer und sicherer als das Aufbrechen des Eies von oben her [,] wie es neuerdings wiederum Moleschott und Dursy empfehlen. [↑](#footnote-ref-1)
2. Remak, der die Methode der Keimabhebung mittelst einer Glasplatte meines Wissens zuerst angewendet

   hat, benutzte zur Reinigung frisches Krebsblut, das, wie ich selbst bei ihm sah, den Zweck vortrefflich erfüllt. Erdl empfiehlt warmes Salzwasser, auch Moleschott und Dursy in ihren neueren Aufsätzen scheinen kein besseres Untersuchungsmedium als circa 1% Salzwasser zu kennen. Das vortreffliche Schultze'sche Medium vereinigt die Zweckmässigkeit der ersteren mit der Bequemlichkeit der letzteren Methode. [↑](#footnote-ref-2)
3. Ich habe im Ganzen die obige Erhärtungsmethode wenig variirt, weil sie mir für die, zunächst vorgesteckten Ziele vortreffliche Dienste leistete. Für specielle Fragen, hauptsächlich für genauere histologische Studien werden sicherlich andere Erhärtungsmethoden wie z.B. die Chromsäurepräparate, und die Goldpräparate sich noch geeigneter erweisen; so habe ich mit Goldchlorid Präparate gewonnen , die vor den Osmiumpräparaten durch schärferes Hervortreten der Zellengränzen sich auszeichneten, die im Uebrigen aber lange nicht dieselbe Zierlichkeit besassen wie diese. [↑](#footnote-ref-3)
